# Experimental Validation of an Inductive System for Magnesium Level Detection in a Titanium Reduction Reactor

**DOI:** 10.3390/s20236798

**Published:** 2020-11-28

**Authors:** Nico Krauter, Sven Eckert, Thomas Gundrum, Frank Stefani, Thomas Wondrak, Ruslan Khalilov, Ivan Dimov, Peter Frick

**Affiliations:** 1 Helmholtz-Zentrum Dresden-Rossendorf, Bautzner Landstr. 400, D-01328 Dresden, Germany; s.eckert@hzdr.de (S.E.); th.gundrum@hzdr.de (T.G.); f.stefani@hzdr.de (F.S.); t.wondrak@hzdr.de (T.W.); 2 Institute of Continuous Media Mechanics, Russian Academy of Sciences, Ural Branch, 614013 Perm, Russia; khalilov@icmm.ru (R.K.); dimov.i@icmm.ru (I.D.); frick@icmm.ru (P.F.)

**Keywords:** Kroll process, numerical simulation, inductive measurements, titanium, level detection

## Abstract

In order to precisely determine the magnesium level in a titanium reduction retort by inductive methods, many interfering influences have to be considered. By using a look-up-table method, the magnesium level can be reliably identified by taking into account the interfering effects of the titanium sponge rings forming at the walls with their unknown geometrical and electrical parameters. This new method uses a combination of numerical simulations and measurements, whereby the simulation model is calibrated so that it represents the experimental setup as closely as possible. Previously, purely theoretical studies on this method were presented. Here, the practical feasibility of that method is demonstrated by performing measurements on a model experiment. The method is not limited to the production of titanium but can also be applied to other applications in metal production and processing.

## 1. Introduction

Titanium is widely used for the construction of highly stressed parts of aircrafts [1] and power plants, as well as in many consumer products [2]. Its most outstanding properties include high specific strength and very good corrosion resistance [3]. While it can be produced by different thermo-chemical or electrochemical reduction processes [4], the commercial production is dominated by the Kroll process [5]. In that process, liquid titanium tetrachloride (TiCl_4_) is reduced to solid titanium sponge by slowly pouring it onto liquid magnesium. During this exothermic reaction

TiCl_4_ + 2 Mg → Ti + 2 MgCl_2_, (1)

the temperature, reaction rate, and formation of lower chlorides (TiCl_2_ and TiCl_3_) have to be carefully monitored and controlled in order to optimize the titanium yield and quality [6]. The reaction is also affected by the complex two-phase flow of Mg and MgCl_2_ within the reactor, which can be influenced by changing the operation regime of the heaters that are located outside of the reactor [7,8]. Efficient heating is also important for minimizing the energy consumption of the whole process. An overview of current electromagnetic industrial heating applications can be found in [9]. A promising approach to increase the overall efficiency of the process is to use a waste heat recovery system [10,11].

**Figure 1:**
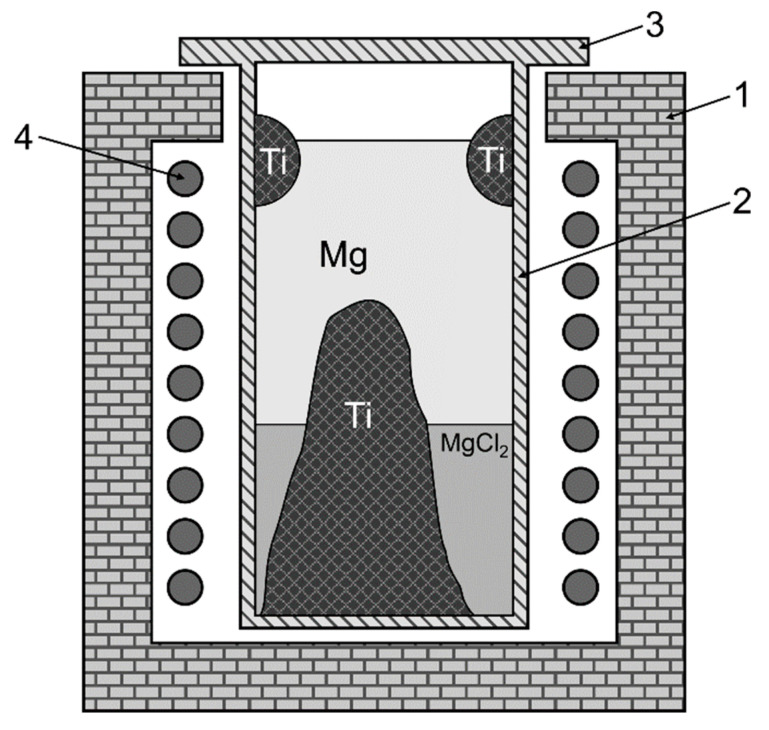
Schematic illustration of a titanium reduction cell. Within a furnace (**1**), the reactor (**2**) is situated which is covered by a lid (**3**). There are 9 heaters (**4**), a few of the uppermost ones will be used as excitation coils. These coils are connected to a current source which provides currents of varying frequency.

Despite significant progress in the numerical simulations of metallurgical processes in general [12], a reliable on-line monitoring of process parameters is in many cases still indispensable. The TiCl_4_ used in the Kroll process has to be handled with particular care, and contact with water has to be prevented because of the strong exothermic reaction between TiCl_4_ and water resulting in the production of hydrochloric acid that can be harmful to workers at the plant when inhaled in its aerosolized form [13]. Unfortunately, the conditions within the reduction retort (see Figure 1), with temperatures between 900 °C and 1050 °C [14], prevent the internal installation of measurement equipment inside the retort. One particularly important parameter to be monitored is the position of the upper surface of the liquid magnesium. There have already been various attempts to use inductive measurement techniques for the estimation of the Mg level from outside of the retort [15,16,17]. However, one obstacle for inductive measurement techniques is the formation of titanium sponge not only—as desired—at the bottom of the retort but also at the walls. Increasing in size during the reduction process, these electrically conducting sponge rings (SR) interfere more and more with the inductive measurements. The geometrical parameters of the SR, and even their electrical conductivity which partly depends on its porosity, are unknown during the production process and change over time. As the existence of the SR can disturb the inductive measurements significantly, an appropriate compensation of this effect is needed for accurate inductive measurements of the magnesium level.

Previously, a new method for the robust and reliable determination of the Mg level was proposed, which takes into account the effects of the SR [18,19]. The sensing system uses the existing heaters as excitation coils and one detection coil on top of the retort. In a preprocessing step, the induced voltage in the detection coil is calculated for all possible parameter combinations of the Mg level and the location, size, and electrical conductivity of the SR. As only one excitation coil is active at a given time, multiple calculations are performed for each parameter combination. The resulting voltages are stored in a look-up table (LuT). For a given measured set of induced voltages, the parameter combination with the smallest deviation stored in the LuT represents the most likely conditions in the retort. In [18], it was shown numerically that by using multi-frequency excitation and considering the position, size, and conductivity of the titanium SR as unknown parameters, the accuracy for the Mg level detection can be significantly increased compared to previous attempts. The usefulness of similar multi-frequency techniques had previously been evidenced for the inductive determination of metallic plate thicknesses [20] and the identification of conductivity profiles [21].

In this paper, the practical feasibility of this multi-frequency measurement procedure will be demonstrated on a small model of a titanium reduction retort. The liquid magnesium is represented by an aluminium cylinder, while the varying sizes and conductivities of the SR are modeled by metal rings. Taking all geometric and material parameters of the experimental setup into account, the LuT is precomputed using a 2D numerical model. The applicability of the new method will be demonstrated by measuring and analyzing the induced voltages for different combinations of Mg level and SR parameters.

After a short description of the LuT procedure, the experimental model as well as the corresponding numerical model will be presented in detail. The main section of the paper describes the results of the measurements and the comparison with the numerical simulation. The paper concludes with a discussion of possible improvements and further application fields of the technique.

## 2. Materials and Methods

### 2.1. Look-Up Table Method

Determining the magnesium level by an inductive method is a highly non-trivial inverse problem because the induced voltages are influenced by various, partly unknown geometrical and electrical parameters. In order to reliably identify the magnesium level within the retort, a large number of forward numerical simulations for certain parameter combinations are performed, and the resulting induced voltages are stored in a LuT. By using multiple excitation coils that are located at several vertical positions around the retort as well as different frequencies of the excitation current, the magnetic fields penetrate into different regions of the experimental setup, resulting in a change of the induced voltage at the detection coil. Only one excitation coil is active at a given time. At lower frequencies, for example, the magnetic fields can penetrate deeper into the liquid Mg and the SR, thus providing additional information for the estimation of the unknown parameters. At higher frequencies, the penetration depth is reduced because of the skin effect, which provides more information about interfaces between domains with different electrical conductivities. Provided that the numerical simulation model is accurate enough in describing the experimental setup, it becomes possible to identify an unknown parameter combination by comparing a set of measured induced voltages with the corresponding numerical values stored in the LuT. By using different excitation coils and frequencies, the accuracy of the parameter estimation can be increased. However, the use of frequencies of the same order of magnitude or excitation coils that are too close together is not recommended since those additional data are too similar and therefore add little to the accuracy of the parameter estimation. The very parameter combination within the LuT whose simulated induced voltages have the lowest deviation from the measured voltages has the maximum likelihood to represent the correct parameter combination. This deviation is expressed by some appropriately constructed mean squared error (MSE) ${\tilde{\alpha }}^{2}$. By calculating the MSE for every entry in the LuT, the unknown parameter combination can be identified simply by searching for the lowest MSE. In [18,19], it was demonstrated that this method can provide quite accurate and robust results, even when the measured voltages are noisy. In the following, the practical feasibility of this method will be validated in a model experiment.

### 2.2. Experimental Setup

**Figure 2:**
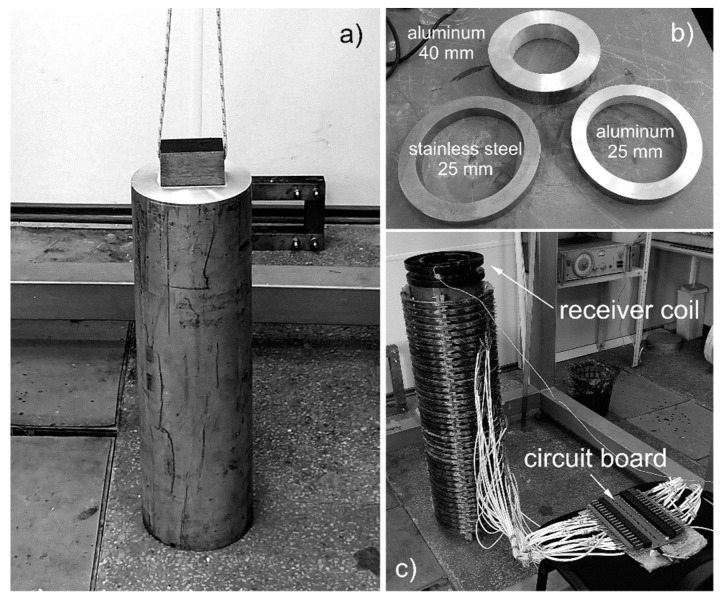
Measurement setup: (**a**) aluminum cylinder; (**b**) metal rings made of stainless steel and aluminum; and (**c**) full setup including receiver coil, 38 emitting coils, and stainless steel channel with aluminum cylinder inside.

For inductive measurement techniques, the electrical conductivity and permeability of the materials are the most important parameters. Because of the high temperatures which lie well above the Curie point of iron, µ_r_ = 1 can be assumed for all materials of the real reduction retort. Liquid Mg has an electrical conductivity of 3.6 MS/m, the Ti sponge has, depending on its porosity, a conductivity between 1 and 2 MS/m. Compared to Ti, the conductivity of TiCl_4_ is negligible. In our mock-up experiment, the wall of the retort is represented by a stainless steel tube, and the liquid magnesium is replaced by a solid aluminum cylinder which can be moved vertically in order to mimic the changing surface level (see Figure 2a). The titanium SR that is usually forming on the inner wall of the retort is represented by three different metal rings, two of which are made of aluminum with quadratic cross sections of 40 mm and 25 mm side length, and one made of stainless steel with a cross section side length of 25 mm (see Figure 2b). This allows to investigate how the measurements are influenced by SR of different sizes and electrical conductivities. The distance between the aluminum cylinder and the metallic rings can be adjusted by inserting non-conductive plastic discs between them. The stainless steel wall is surrounded by 38 identical and equidistant emitting coils that generate alternating magnetic fields which induce eddy currents within the retort (see Figure 2c). All materials of the mock-up have a relative permeability of µ_r_ = 1. As the real retort is made of stainless steel, it is also used for the retort of the mock-up. The use of aluminum to represent the liquid Mg was mainly motivated by its low weight and cost, and its easy machinability. The much higher electrical conductivity (26 MS/m) of Al compared to liquid Mg can be factored in by an appropriate scaling of the excitation frequency to achieve comparable relative skin depths. What is more important is to correctly mimic the ratio of the conductivity of the interfering SR to that of Mg. Aluminum rings represent highly conductive sponges, whereas stainless steel rings represent sponges with very low conductivity. As the influence of the SR on the sensor signal is always a combination of its conductivity and size, two sizes of aluminum rings were produced to represent different sponge rings. A small, highly conductive ring has a similar influence as a larger, slightly less conductive ring. The three rings used for the mock-up can cover the most important parameter combinations that are expected in the real production process.

During the experiment, only one emitting coil is active at a given time. With view on the limited measurement options in the real titanium production process, only one, but highly sensitive, receiver coil is placed on the top of the experiment. It is used for measurements of the total magnetic field that is generated by the emitting coils and influenced by the induced eddy currents. These currents change in dependence on the geometrical and electrical parameters of the respective experimental setup. Measurements are performed for certain experimental parameter combinations of the position of the aluminum cylinder surface *h*_Al_, the vertical position *h*_s_ of the SR, its cross section side length *a*_s_, and its electrical conductivity *σ*_s_. After the induced voltages in the receiver coil for each emitting coil have been recorded, a new experimental parameter combination is chosen. A total of 40 different parameter combinations were used in the experiment (see Table 1).

In order to enhance the accuracy of this method, multiple frequencies f of the excitation current are used. Depending on the frequency, the penetration depth of the magnetic field into the conductive components of the setup is changed and allows for more detailed information regarding the geometrical and electrical parameters to be extracted from the measured voltages. A circuit board is used to iterate through all of the 38 emitter coils and frequencies.

**Figure 3:**
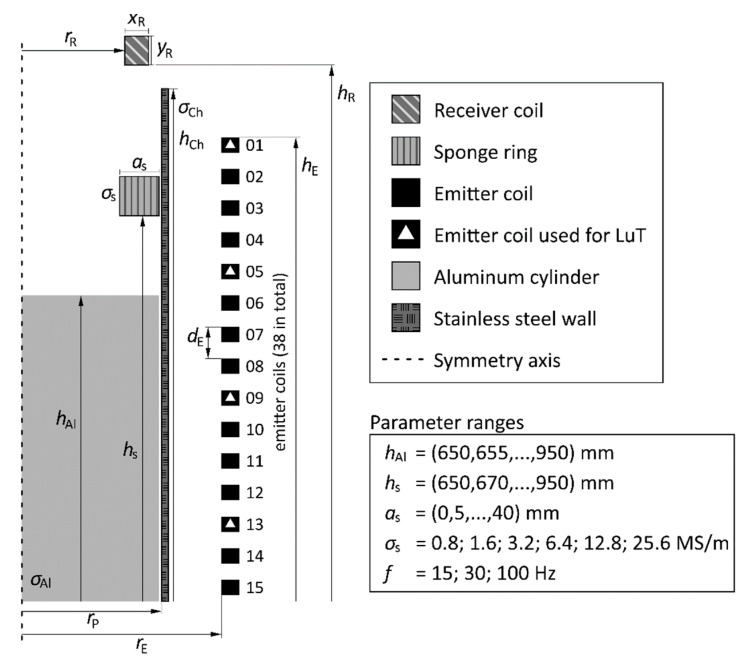
Upper part of the 2D COMSOL simulation model and the corresponding parameter ranges for the numerical forward calculation.

As in [9], COMSOL Multiphysics v5.5 with the magnetic and electric fields interface was utilized for the calculation of the LuT. Because of the geometric arrangement of the model experiment, an axisymmetric 2D simulation model was chosen to represent the measurement setup. Compared to a full 3D simulation, this has the advantage that a considerable amount of calculation time can be saved. The upper part of the simulation model is delineated in Figure 3. The LuT contains the magnitudes and phase shifts of the induced voltages for all experimental parameter combinations of *h*_Al_, *h*_s_, *a*_s_, *σ*_s_, as computed for three frequencies and four emitter coils. Anticipating the typical positions of the heater coils in a specific titanium plant (which might easily be “misused” also as emitter coils), for our LuT only the emitter coils C1, C5, C9 and C13 are used, although the voltages for all coils were recorded both in simulation and measurement. In most cases, the use of more than four emitter coils for this setup only yields marginal benefits because the corresponding induced voltages are very similar and provide no additional information for the parameter estimation. In total, the induced voltages for 60.381 different parameter combinations were calculated which took 20 h on a computer with a Core i5-8500 processor and 16 GB of RAM.

The magnitudes and phase shifts of the induced voltage were measured with a lock-in amplifier. This allows for very accurate magnitude and phase measurements as it only considers signals with the same frequency as the excitation current, while electromagnetic disturbances with other frequencies are filtered out almost completely. A current controlled power supply was used to ensure excitation currents with a constant amplitude even in case of slight variations in the excitation coils’ resistance. All connections to and from the coils were made of twisted pair wires to further reduce the influence of stray magnetic fields on the measured voltage.

The geometrical and electrical parameters that were used for the calculation of the LuT are indicated in Table 2. Due to geometric constraints of the experiment, in which a submerged sponge ring is not possible (i.e., *h*_Al_ < *h*_s_), not all of the combinations had to be computed in the LuT. Most of the parameters are based on the dimensions of the model experiment, but for some parameters, like the electrical conductivities of the stainless steel channel *σ*_Ch_ and the aluminum (Alu) *σ*_Al_, the exact values were unknown. Therefore, a calibration of the simulation model was performed in order to fix those geometrical, electrical, and material parameters that were not perfectly known beforehand. By performing measurements for some generic cases and comparing them to the output of the numerical model, these parameters for the simulation model were adjusted to best represent the experimental setup. This also takes the impedance change between empty and filled mock-up into account. The values of *σ*_Ch_ and *σ*_Al_, as well as some geometrical parameters, were obtained by comparing the simulation results with the measurement results of two distinct cases. In the first case, a completely empty channel was used, so that *σ*_Ch_ and the exact vertical position of the receiver coil *h*_R_ could be calibrated without the interference of the aluminum cylinder. This calibration turned out to be necessary, because even a displacement of a few hundred micrometers can significantly influence the accuracy of the LuT method. In the second case, the aluminum cylinder was put on a fixed position at *h*_Al_ = 845 mm, which allowed for an estimation of *σ*_Al_. The final calibrated values are listed in Table 2. In the numerical simulation model, the experiment is put in the center of a large virtual cylinder that consists of air with a height of *h*_air_ and a radius of *r*_air_, which is necessary to allow for a correct modeling of the magnetic field lines. If the volume of air around the experiment is chosen too small, a large error for the induced voltage within the receiver coil was obtained.

## 3. Results

For the evaluation of the measurement results, the previously calculated LuT was used. As it contains the magnitudes and phases of the receiver voltages for a large number of parameter combinations, it can be used to identify the actual parameter combination by its measured voltages. This is achieved by comparing the measured voltages (magnitudes and/or phases) of a single measurement to all voltages within the LuT. The actual parameter combination in the experiment can then be identified by finding the corresponding parameter combination in the LuT that has the lowest deviation from the measured voltages. In other words, a low deviation between the LuT values and measurement values indicates a high likelihood of matching parameter combinations between LuT and measurement.

### 3.1. Calculation of the Weighted Mean Squared Error

For each of the *k* parameter combinations of the numerical simulation, the weighted mean square error (MSE) ${\tilde{\alpha }}_{k}^{2}$ is calculated. The weighted MSE of the voltage magnitude is called ${\tilde{\alpha }}_{Mk}^{2}$, for the phase it is ${\tilde{\alpha }}_{Pk}^{2}$. They are calculated from the differences of the measured voltage magnitudes *V*_Meas_ (or phases *P*_Meas_) and all corresponding values *V*_LuT,*k*_ (or *P*_LuT,*k*_) from the LuT. Each *V*_Meas_ (and *P*_Meas_) comprises a number of separate values which is equal to the product of the number of frequencies *n*_f_ and the number of emitter coils *n*_c_ which are, in the presented case, *n*_f_ = 3 and *n*_c_ = 4. The two types of MSE are defined as follows:

(2) $${\tilde{\alpha }}_{Mk}^{2}=\frac{1}{n_{c}n_{f}}\overset{n_{f}}{\underset{i=1}{\sum }}\overset{n_{c}}{\underset{j=1}{\sum }}{\left(\frac{1}{v_{i,j}}\left(V_{\mathrm{Meas},i,j}-V_{\mathrm{LuT},k,i,j}\right)\right)}^{2}\;\;\;\;\;\;\;\;\;\;k=1\ldots 60,381$$

(3) $${\tilde{\alpha }}_{Pk}^{2}=\frac{1}{n_{c}n_{f}}\sum_{i=1}^{n_{f}}\sum_{j=1}^{n_{c}}{\left(\frac{1}{p_{i,j}}\left(P_{\mathrm{Meas},i,j}-P_{\mathrm{LuT},k,i,j}\right)\right)}^{2}\;\;\;\;\;\;\;\;\;\;k=1\ldots 60,381.$$

Every individual square error in Equations (1) and (2) is weighted by $v_{i,j}$ and $p_{i,j}$ appropriately in order to compensate for the influence of various effects, like the coil distance and frequency, on the overall MSE. Without such a weighting, the closest emitter coil and the highest frequency would have the largest impact on the total MSE of the voltage magnitudes, whereas emitter coils that are farther away from the sensor coil, or measurements at lower frequencies, would have a reduced or even negligible influence on the total MSE. To avoid this effect, the square errors for each emitter coil and frequency are weighted using the variance—taken over all respective parameter combinations—of the simulated voltage magnitudes or phases. When dividing the respective square errors by those variances, all square errors are rescaled in a balanced manner according to how much they are changing over the whole experimental parameter range. The corresponding standard deviations $v_{i,j}$ and $p_{i,j}$ for the voltages and phases, as used in Equations (1) and (2), respectively, are given in Table 3 and Table 4.

The dimensionless MSEs ${\tilde{\alpha }}_{Mk}^{2}$ and ${\tilde{\alpha }}_{Pk}^{2}$ can be evaluated separately or in the combination

(4) $${\tilde{\alpha }}_{Ck}^{2}=\frac{{\tilde{\alpha }}_{Mk}^{2}+{\tilde{\alpha }}_{Pk}^{2}}{2}.$$

Indeed, in previous numerical simulations [9,10] it had been observed that the combination ${\tilde{\alpha }}_{Ck}^{2}$ of the MSE for magnitude and phase yields more reliable results in the majority of cases.

After calculating the MSE for all *k* parameter combinations, the unknown parameter combination can be estimated by simply finding the lowest MSE, because the parameter combination with the lowest MSE has the maximum likelihood to represent the unknown parameter combination. Further investigations have shown that the reliability of the results can be judged by calculating the values for multiple parameter combinations with a low MSE. For the analysis of this experiment, parameter combinations with the 50 lowest MSEs are used to estimate the respective parameters.

### 3.2. Evaluation of Results

For the evaluation of the results, the MSE of the voltage magnitude ${\tilde{\alpha }}_{M}^{2}$, the phase ${\tilde{\alpha }}_{P}^{2}$ and their combination ${\tilde{\alpha }}_{C}^{2}$ were calculated for all *k* parameter combinations that were studied in the numerical simulation. Then, the parameters *h*_Al,Calc_, *h*_s,Calc_, *a*_s,Calc_, and *σ*_s,Calc_ were estimated by calculating the mean value of the respective 50 parameter combinations with the lowest MSE. From the experiment, the correct parameters *h*_Al,Exp_, *h*_s,Exp_, *a*_s,Exp_, and *σ*_s,Exp_ are known (or were measured). In order to evaluate the quality of these estimations, the differences

(5) $$\Delta h_{\mathrm{Al}}=h_{\mathrm{Al},\mathrm{Exp}}-h_{\mathrm{Al},\mathrm{Calc}}$$

between the known values (here *h*_Al,Exp_ taken as a specific example) and the estimated values (*h*_Al,Calc_ again as an example) are considered. In order to evaluate the average Δ*h*_Al_ for *n* measurements, the mean deviation *λ*(*h*_Al_) is introduced as follows:

(6) $$\lambda (h_{\mathrm{Al}})=\sqrt{\frac{1}{n}\overset{n}{\underset{i=1}{\sum }}\Delta h_{\mathrm{Al},i}{}^{2}}.$$

**Figure 4:**
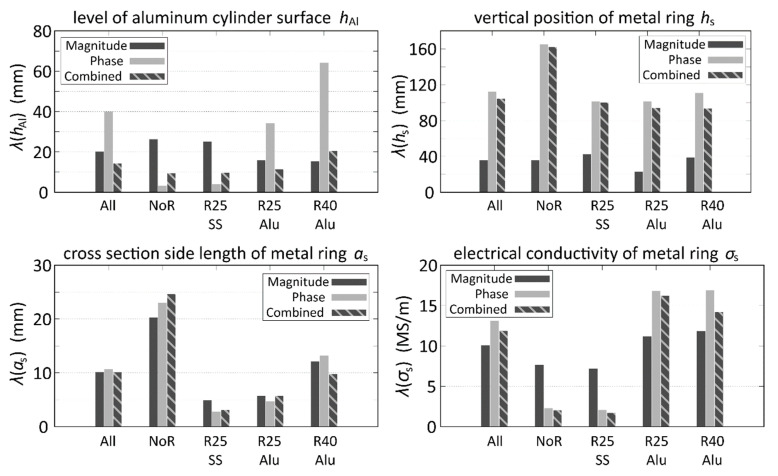
Mean deviation λ of the 4 estimated parameters in dependence on the metal ring properties. A total of 5 cases are displayed: All measurements (All), measurements without metal rings (NoR), measurements with the stainless steel ring of 25 mm cross section side length (R25SS), the aluminum ring (R25Alu), and the 40 mm aluminum ring (R40Alu).

**Figure 5:**
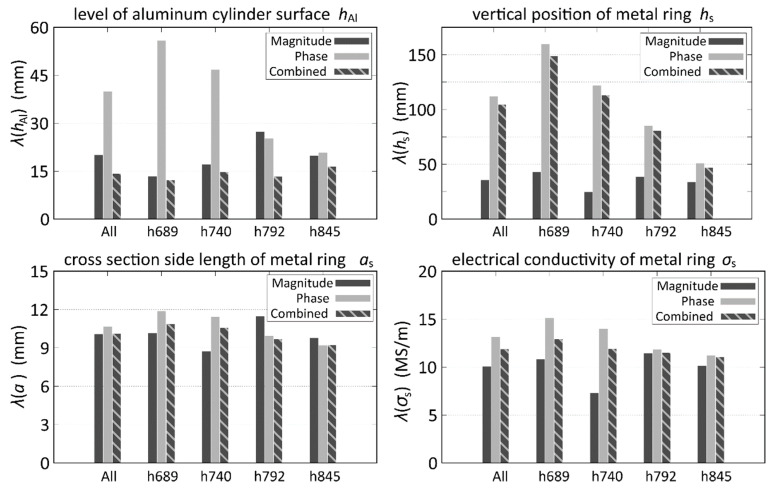
Mean deviation λ of the 4 estimated parameters in dependence on the surface level *h*_Al,Exp_ of the aluminum cylinder. A total of 5 cases are displayed: All measurements (All) and four different *h*_Al,Exp_ of 689 mm, 740 mm, 792 mm and 845 mm.

In the following, the resulting *λ* are displayed in two different ways in order to demonstrate the specific influences of certain variations on *λ* for each of the four parameters to be determined: In Figure 4, the influence of the metal rings is displayed, and in Figure 5, the influence of the position of the aluminum cylinder surface on the mean differences is displayed.

From Figure 4, where the 40 experimental parameter combinations are divided according to which metal ring was used in the respective measurement, some general conclusions can be drawn: Averaged over all parameters it is most suitable to use the combined MSE of magnitude and phase in order to increase the accuracy of the parameter estimation of *h*_Al_. For the rings with a high *σ*_s_, the expected behavior of *λ*(*h*_Al_) can be observed, which is generally increasing with higher *a*_s_ and *σ*_s_. It can also be seen that for cases without rings or for rings with a low electrical conductivity, the phase MSE is very small. On the other hand, it becomes quite large for the aluminum rings with a high *σ*_s_. For the estimation of *h*_s_, the MSE of the voltage magnitude yields the best results for all cases. Note also that the parameter estimation for the remaining parameters, i.e., *a*_s_ and *σ*_s_, does not yield very accurate results, which means that these parameters can only be roughly estimated. The main reasons for this behavior is the intrinsic non-uniqueness of the underlying inverse problem, which results in an ambiguity of attribution: since a certain induction effect can either be due to a thin ring with high conductivity or a thick ring with lower conductivity. However, in most cases this non-uniqueness concerning *a*_s_ and *σ*_s_ is not detrimental for the determination of *h*_Al_, which is, after all, the most relevant quantity.

Another interesting effect can be observed in Figure 5. Here, the 40 parameter combinations of the experiment are divided according to the level of the aluminum cylinder surface. There is no clear correlation between the actual position of the surface and *λ*(*h*_Al_), at least for the combined MSE, which again yields the best results for the estimation of *h*_Al_. For *h*_s_, the MSE of the magnitude performs much better than the combined or the phase MSE. There seems to be no significant influence of *h*_Al,Exp_ on *λ*(*a*_s_) and *λ*(*σ*_s_), although they are slightly decreasing with higher *h*_Al,Exp_.

### 3.3. Detailed Analysis of Results

**Figure 6:**
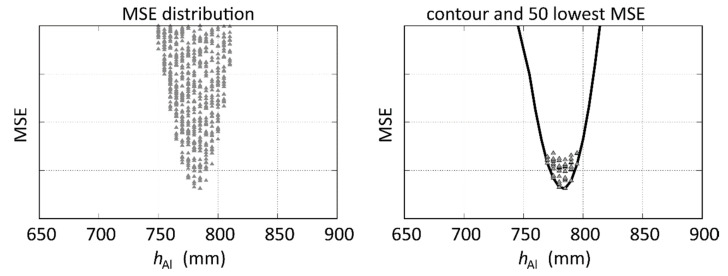
Example for extracting the contour from the MSE distribution and the location of the 50 lowest MSE for the experiment with *h*_Al,Exp_ = 792 mm, *h*_s,__Exp_ = 876 mm, *a*_s,__Exp_ = 25 mm, *σ*_s,__Exp_ = 1.3 MS/m.

In this section, the distribution of MSE will be analyzed in more detail in order to identify the reasons for deviations and the advantages of combining magnitude and phase data. For that purpose, the raw MSE distribution will be converted to a contour (see Figure 6) to simplify the analysis. In the example in Figure 6, the lowest 50 MSE which are used to calculate the average value of the parameters are also displayed. As we see, the correct value of *h*_Al,Exp_ = 792 mm is well identified in the minimum of the parabola-shaped lower part of the contour.

**Figure 7:**
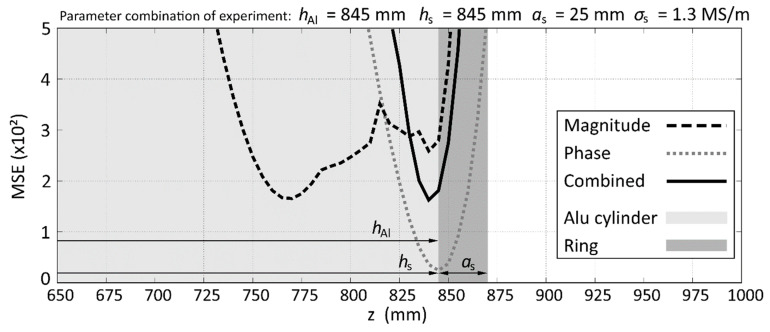
Contours for a case of a stainless steel ring situated close to the surface of the aluminum cylinder.

In the following figures, the contours for voltage, phase, and their combination, as well as the location of the aluminum cylinder and the rings are displayed exclusively for the most interesting parameter *h*_Al_. The investigation of the accuracy of the Al-level is started with a low conducting SR. Figure 7 shows the contours for the case of a stainless steel ring close to the surface of the aluminum cylinder. It can be seen that the individual MSE of magnitude and phase already have a local minimum close to the correct *h*_Al_, but there are also additional minima that are not close to the correct position. However, the combination of magnitude and phase has a clear global minimum at the correct *h*_Al_, while the other minima are shifted to higher MSE. There are also few cases where the individual phase or magnitude MSE is more accurate than their combination, but, in general, using the combination of magnitude and phase MSE yields more reliable and accurate results.

The other measurements for which a stainless steel ring was used also yielded very good results. For those cases taken separately, a mean deviation *λ*(*h*_Al_) of 9.6 mm for the estimation of *h*_Al_ was achieved. This confirms the expectation that a sponge ring with a very low electrical conductivity also has a low impact on the accuracy of this measurement technique.

**Figure 8:**
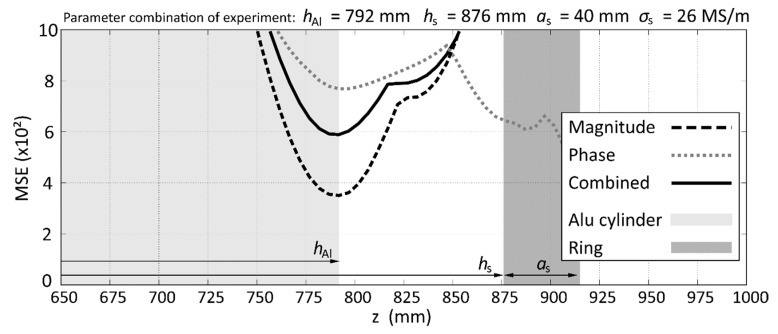
Contours for the case of an aluminum ring which represents the SR situated far away from the surface of the aluminum cylinder.

If the electrical conductivity of the SR is increased to 26 MS/m, which is equal to the conductivity of the aluminum cylinder, the measurement of *h*_Al_ becomes more difficult. Figure 8 shows the case for a large, highly conductive ring. As long as the ring is not located directly above the surface of the aluminum cylinder, the correct *h*_Al_ can still be identified with reasonable accuracy. In this example, it can also be seen that the MSE of the magnitude already indicates the correct *h*_Al_, whereas the MSE of the phase has multiple minima. The combined MSE for magnitude and phase gives a very clear global minimum at the correct location. Regarding *λ*(*h*_Al_), this is also the case with the lowest deviation of only 1.1 mm to the actual position of the aluminum surface.

**Figure 9:**
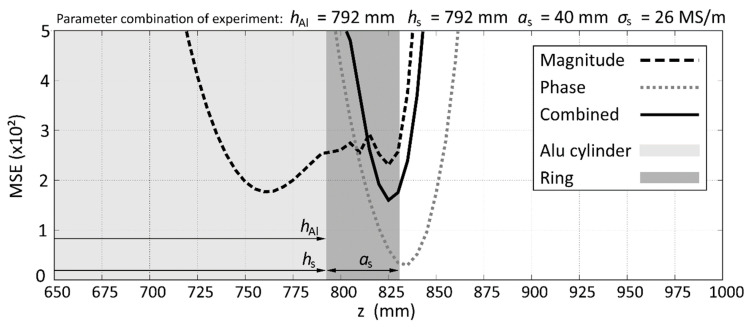
Contours for the case of an aluminum ring situated directly above the aluminum cylinder.

Expectedly, the most difficult cases for determining *h*_Al_ are those with a highly conductive ring directly above the surface. For these cases, there are typically multiple minima in the MSE distribution, as seen in Figure 9. They are caused by parameter combinations that have a very similar influence on the induced voltages. Unfortunately, the deepest minima are found at the wrong position of the upper edge of the ring. This is not surprising, as a large, highly conductive ring directly above the aluminum surface behaves electrically similar to the case without ring but with a slightly higher position of the aluminum surface. Especially at high frequencies, the penetration depth of the magnetic fields is not enough to allow a distinction between these very similar cases. To resolve this issue, the use of even lower frequencies than 15 Hz could be helpful. With this example it can be seen that the choice of parameter ranges of the numerical simulation also has an influence on the final results and number of minima in the MSE distribution, when there are two or more parameter combinations that have similar results. The cases with a high *h*_Al_ and a low *h*_Al_ with high *a*_s_ and *σ*_s_ are very hard to distinguish. Fortunately, in the real titanium production process, the sponge ring typically has a lower electrical conductivity than the liquid metal. Accordingly, in our previous investigations [9,10] of this method, “wrong” minima in the MSE distribution could not be observed because the sponge ring was assumed to have 50% or even lower electrical conductivity than the liquid metal.

### 3.4. Discussion of Errors

As mentioned previously, some of the simulation parameters (like the electrical conductivity of the aluminum) are not exactly known. Therefore, a calibration of the simulation model was performed for two specific experimental setups. The calibrated parameters were chosen in a way to maximize the agreement between numerical simulation and experiment. Without knowing the real values for these parameters, the resulting error of the calibrated parameters cannot be estimated. For the simulation model, which is only an idealized 2D representation of the experimental setup, certain simplifications have been made. This can also be a source for additional errors. The presence of multiple minima in the MSE distribution, which is caused when two or more parameter ranges have similar effects on the induced voltages, also hampers the correct parameter estimation, especially in combination with measurement errors and other systematic errors. In the real experiment, there is no total axisymmetry, the emitter coils might not be perfectly equidistant and even when some distances are off by a few millimeters, the induced voltages change significantly. During the experiments, it could be observed that the whole setup is very sensitive to changes in the distance between the coils. Another point to consider for the practical application of this method is the influence of the surrounding electrically conductive and/or ferromagnetic objects in the vicinity of the measurement setup. All this will require careful calibration on an empty device.

## 4. Discussion

In this experiment, the practical feasibility of the LuT method for determining the level of liquid magnesium in a laboratory mock-up of a titanium reduction retort was demonstrated. The accuracy of the LuT method can be improved significantly when weighting the calculated MSE according to the proposed method which compensates for the influence of the distance between emitter coils and the different excitation frequencies. By using the MSE of the voltage magnitude, phase, and their combination it was demonstrated that the combined approach is, in general, the optimal choice. This corresponds to the findings from previous studies on this LuT method because the number of information to determine the parameter combination is essentially doubled. Considering all cases, the position of the surface of the aluminum cylinder can be obtained with an average deviation of 14.2 mm. When only considering the parameter combinations without or with lowly conductive rings, the standard deviation is reduced to 9.6 mm for the combined MSE and to 3.7 mm when using the phase MSE. Due to the intrinsic non-uniqueness of the underlying inverse problem, connected with an ambiguity of attribution, the average deviation for the other parameters like the position, size, and electrical conductivity of the metal ring is quite large, so that they cannot be identified with reasonable accuracy.

The case of a highly electrically conductive ring situated close to the aluminum surface remains a challenge for an accurate determination of *h*_Al_ as it decreases the accuracy for the estimation of the location of the aluminum surface. As in the technological application of this method the rings have an, at least by a factor of 2, lower electrical conductivity than the liquid metal, it might be much easier to identify the correct position of the liquid metal surface compared to the cases of the model experiment, where aluminum rings with the same electrical conductivity as the cylinder are used. Using lower frequencies than 15 Hz in future experiments might therefore increase the accuracy of this method because the penetration depth of the magnetic fields would be increased, which allows an improved distinction between two parameter combination with similar electrical behavior. Using more than four emitter coils could also yield some slight benefits regarding the measurement accuracy. Other methods for the inductive detection of liquid metal surfaces strongly rely on the absence of interfering conductive bodies near the sensor. In most cases, like, for example, in [22], the liquid metal surface can be identified with reasonable accuracy and time resolution by using one excitation and detection coil at one given frequency. However, these techniques quickly run into problems, as soon as there are interfering influences. Although the multi-coil/multi-frequency approach that was presented in this paper is more complex in comparison, it can also compensate for these disturbances by providing more detailed information about the conditions inside the retort, which enable more accurate measurements of the position of the surface.

## 5. Conclusions

In this paper, the practical viability of the previously proposed LuT method for determining the level of liquid magnesium during the production of titanium via the Kroll process has been demonstrated by performing and evaluating experiments on a mock-up of a titanium reduction retort. Although the presence of titanium sponge rings is strongly interfering with the measurements, it has been shown that this problem can be overcome by applying this LuT method to the problem. This combination of measurements and a finely tuned numerical model of the setup is not only able to identify the level of the liquid magnesium almost in real time but it also provides a measure for the quality of the results by presenting a few hundred of the most likely parameter combinations that best fit the measurements. At the model experiment, a combination of different (solid) metals was used to represent the typical components of the reduction reactor and the materials it contains. A problem that remains is the presence of a large, highly conductive sponge ring. In this case, which is however unlikely to appear during the real production process of titanium, the level of magnesium cannot be reliably identified. The next step will be the application of this measurement technique to the real titanium production process.

The method could also be adapted to other problems where interfaces between differently conducting fluids are to be continuously inspected, such as in liquid metal batteries [23], where a weakly conducting liquid electrolyte is sandwiched between a lower and an upper liquid metal layer.

## Figures and Tables

**Table 1:** Overview of the 40 parameter combinations that were used in the experiment.

*h*_Al,Exp_ (mm)	*h*_s,Exp_ (mm)	*a*_s,Exp_ (mm)	*σ*_s,Exp_ (MS/m)	*h*_Al,Exp_ (mm)	*h*_s,Exp_ (mm)	*a*_s,Exp_ (mm)	*σ*_s,Exp_ (MS/m)
689	689	without metal ring		792	792	without metal ring	
		25	1.3, 26			25	1.3, 26
		40	26			40	26
	774	25	1.3, 26		876	25	1.3, 26
		40	26			40	26
	887	25	1.3, 26		902	25	1.3, 26
		40	26			40	26
740	740	without metal ring		845	845	without metal ring	
		25	1.3, 26			25	1.3, 26
		40	26			40	26
	824	25	1.3, 26		929	25	1.3, 26
		40	26			40	26
	938	25	1.3, 26		949	25	1.3, 26
		40	26			40	26

**Table 2:** Overview of the simulation parameters.

Parameter	Symbol	Range/Magnitude	Unit
Aluminum cylinder upper edge	*h*_Al_	650–950	mm
Sponge ring lower edge	*h*_s_	650–950	mm
Sponge ring cross section side length	*a*_s_	0–40	mm
Receiver coil inner radius	*r*_R_	78.5	mm
Receiver coil lower edge	*h*_R_	1016	mm
Receiver coil cross section width	*x*_R_	18	mm
Receiver coil cross section height	*y*_R_	22	mm
Emitter coils inner radius	*r*_E_	136.5	mm
Emitter coils upper edge of first coil	*h*_E_	964	mm
Emitter coils offset between adjacent coils	*d*_E_	24.1	mm
Emitter coil cross section width	*x*_E_	13.5	mm
Emitter coils cross section height	*y*_E_	11.7	mm
Stainless steel channel height	*h*_Ch_	996	mm
Stainless steel channel inner radius	*r*_Ch_	107.5	mm
Stainless steel channel wall thickness	*d*_P_	2	mm
Aluminum cylinder outer radius	*r*_Al_	105.5	mm
Air cylinder height	*h*_Air_	4.5	m
Air cylinder radius	*r*_Air_	2.5	m
Excitation current	*I*	1	A
Frequency of excitation current	*f*	15; 30; 100	Hz
Number of turns of receiver coil	*n*_R_	3000	1
Number of turns of emitter coil	*n*_E_	152	1
Electrical conductivity of aluminum cylinder	*σ*_Al_	26	MS/m
Electrical conductivity of metal (sponge) ring	*σ*_s_	0.8–25.6	MS/m
Electrical conductivity of stainless steel	*σ*_Ch_	2.7	MS/m

**Table 3:** Standard deviation $v_{i,j}$ of the receiver coil voltage magnitude for all simulated parameter combinations, for each of the four emitter coils—C1, C5, C9, and C13—and the three excitation current frequencies.

*f*/Hz	C1: *v*/mV	C5: *v*/mV	C9: *v*/mV	C13: *v*/mV
15	120.43	103.06	69.42	25.64
30	421.35	289.32	159.47	52.98
100	2385.59	1339.61	582.16	173.55

**Table 4:** Standard deviation $p_{i,j}$ of the of the receiver coil voltage phase shift for all simulated parameter combinations, for each of the four emitter coils and the three excitation current frequencies.

*f*/Hz	C1: *p*/°	C5: *p*/°	C9: *p*/°	C13: *p*/°
15	3.39	5.93	6.59	4.48
30	4.22	6.67	6.04	4.15
100	4.50	6.14	4.45	3.45
